# Speech and Language Markers of Bipolar Disorder: Challenges and Opportunities

**DOI:** 10.1111/bdi.70119

**Published:** 2026-06-26

**Authors:** Farida Zaher, Jessica Ahrens, Delphine Raucher‐Chéné, Alban Voppel, Lena Palaniyappan

**Affiliations:** ^1^ Department of Psychiatry, Douglas Mental Health University Institute McGill University Montreal Quebec Canada; ^2^ Integrated Program in Neurosciences McGill University Montreal Quebec Canada; ^3^ Department of Psychiatry, Schulich School of Medicine and Dentistry Western University London Ontario Canada; ^4^ Robarts Research Institute, Schulich School of Medicine and Dentistry Western University London Ontario Canada

**Keywords:** acoustics, bipolar disorder, depression, linguistics, mania, natural language processing, speech

## Abstract

**Background:**

Clinicians aspire to predict the emergence of Bipolar Disorder (BD) in a timely manner. To accomplish this, markers reflecting mental states that can be gathered non‐invasively and at large scale are needed. Here, we systematically evaluate evidence relating speech‐based markers to mood states in BD.

**Methods:**

We searched Medline and Google Scholar for all published studies in English up to February 2026 on the use of speech markers in BD. We undertook thematic analysis on abstracts using topic modeling and a qualitative gap analysis to identify potential opportunities for future research.

**Results:**

43 out of 867 studies were included after screening. Topic analysis revealed an emerging focus on mapping mood states to automated speech features. Most studies focused on cross‐sectional *detection* of bipolar mood states, or BD as a diagnosis, rather than the *prediction* of upcoming mood states. Speech features distinguished BD from schizophrenia, depression, and healthy controls. Manic states were characterized by quantifiable measures of pressured speech, derailment, grammatical errors, and word repetition; depressive states by an increased use of personal pronouns, reduced verbal fluency, and speech quantity. Overall, attempts to replicate observations were limited.

**Conclusion:**

Acoustic and lexical‐semantic markers vary with manic, psychotic, or depressive states. At present, the evidence is insufficient for clinical utility in relapse prediction, response monitoring, or diagnosing mixed episodes or state changes in BD. We recommend that future research leverages the growing capabilities of natural language processing through longitudinal and cross‐linguistic studies to strengthen the evidence base and advance the clinical utility of speech markers for BD.

## 1 Introduction

Early detection of impending states in bipolar disorder (BD) can aid in earlier and more accurate intervention, as its presentations—whether states with manic, hypomanic, or depressive features—often mimic phenomenologically related disorders (unipolar depression, non‐affective psychosis, or substance‐induced episodes). The fluctuations in emotions, energy, and cognitive functions associated with BD are often unpredictable and, in some cases, occur repeatedly despite treatment. These repeated recurrences (relapses) are associated with a higher risk of suicide [1, 2], especially in depressed or episodes with mixed features [3]. Thus, reliable prediction of the emerging mood states when they are subtle may allow early detection of both onset and recurrences and thus timely interventions in BD.

While the clinical gains achieved by blood‐ and brain‐based biomarkers have been limited [4, 5], two other objective measures have gathered pace in recent years. The first is digital phenotyping using wearables that detect activity patterns, extensively reviewed elsewhere [6, 7]. The second is the use of speech recordings to detect changes in voice (acoustics) and communication patterns (i.e., the form and content of language). Clinicians routinely use observed speech (i.e., sampling of interpersonal communication) as a critical source of information to rate and measure symptoms. Speech can be economically and non‐invasively obtained and has been hailed as a *biosocial* marker for mental illnesses [8]. We can now objectively quantify clinically pertinent features from speech using automated Natural Language Processing (NLP) approaches. Here, we review the work done to date in BD using various speech‐based markers.

Readily accessible and objective markers of the illness have three key applications in BD. Firstly, in differentiating first episode BD and Major Depressive Disorder (MDD) from non‐affective psychosis. Confident identification on the basis of cross‐sectional assessment of symptoms [2, 9] or cognitive measures [10, 11] alone continues to be a challenge but has important therapeutic and prognostic implications. Secondly, in predicting the emergence of mixed features after remission from a depressive episode. Hypomania is often the first sign of BD in 12%–17% of patients being treated for depression, but is often under‐reported by patients and missed by clinicians [12, 13, 14], increasing the risk for further antidepressant‐related mood destabilization [15, 16]. Third, in identifying episodes with mixed features. The presence of features with opposite polarity during a mood state is increasingly recognized to be more common (states with mixed features [17]) but is often missed in practice, leading to inadequate treatment [18]. Developing readily accessible markers can improve the diagnostic accuracy (psychosis vs. mania), specify clinical states with precision (mixed features, upcoming relapses), and inform us of the likely disease trajectory (unipolar to bipolar transitions, or a misdiagnosis of BD depressive episodes); all of these are well‐known unmet needs in the clinical care of BD [19]. In addition, potential “trait” markers may identify individuals at risk of first episode mania, allowing for early intervention that may have superior outcomes [20, 21].

Language disturbances have been one of the core diagnostic symptoms of both manic and depressive states. As per the DSM‐5, speech in mania is “rapid, pressured, loud and difficult to interrupt…(patients) may talk continuously and without regard for others' wishes, often in an intrusive manner or without concerns for the relevance… sometimes characterized by jokes, puns, amusing irrelevancies, and theatricality” [22]. In depression, speech changes are captured as psychomotor retardation: “slowed speech, thinking, increased pauses before answering; speech that is decreased in volume, inflection, amount, or variety of content, or muteness” [22]. Thus, speech features constitute the diagnostic criteria for mood states, unlike many other digital phenotype markers (e.g., geolocations, step counts, call and text logs, electrodermal hypoactivity), which are only indirectly related to the constructs of depression and mania [23, 24]. Several cognitive measures relevant to BD (e.g., verbal fluency) [25] can also be remotely obtained using digital tools [26, 27], but speech can be sampled in completely naturalistic settings (e.g., during a routine clinical interaction, a social conversation, etc.) and repeatedly acquired for longitudinal studies without invasive or intrusive procedures. This unique combination of construct and ecological validity makes speech markers well positioned for clinical applications in BD.

Natural Language Processing (NLP), a branch of artificial intelligence (AI), combines conventional computational linguistics with speech technologies (e.g., automatic speech recognition) in an attempt to make human language machine usable. By using the inherent hierarchical statistical structure of human language, voice‐related information in speech, and contextual features of discourse, NLP tools allow us to extract acoustic or paralinguistic, syntactic, semantic, sentimental, and pragmatic markers from speech recordings [28, 29, 30]. In effect, clinical NLP can be used to analyze any spoken or written material, by patients or by caregivers. While a review has highlighted NLP's potential for characterizing BD when applied to written text (Electronic Health Records and Reddit/Twitter posts), here we specifically focus on speech markers [31].

We systematically review prior studies examining speech in BD using automated (NLP) approaches and synthesize the features of speech and language that are specific in identifying and predicting the various mood states in BD. Based on a long line of rating‐scale‐based assessments of speech, language, and thought disorders in BD [32, 33, 34], verbal fluency studies [25, 35, 36, 37], and first‐person reports [38], we expected to see (1) illness‐specific and (2) state‐specific speech and language variables in BD, and potentially (3) the use of speech markers to predict impending mood states.

## 2 Methods

### 2.1 Search Process

**FIGURE 1:**
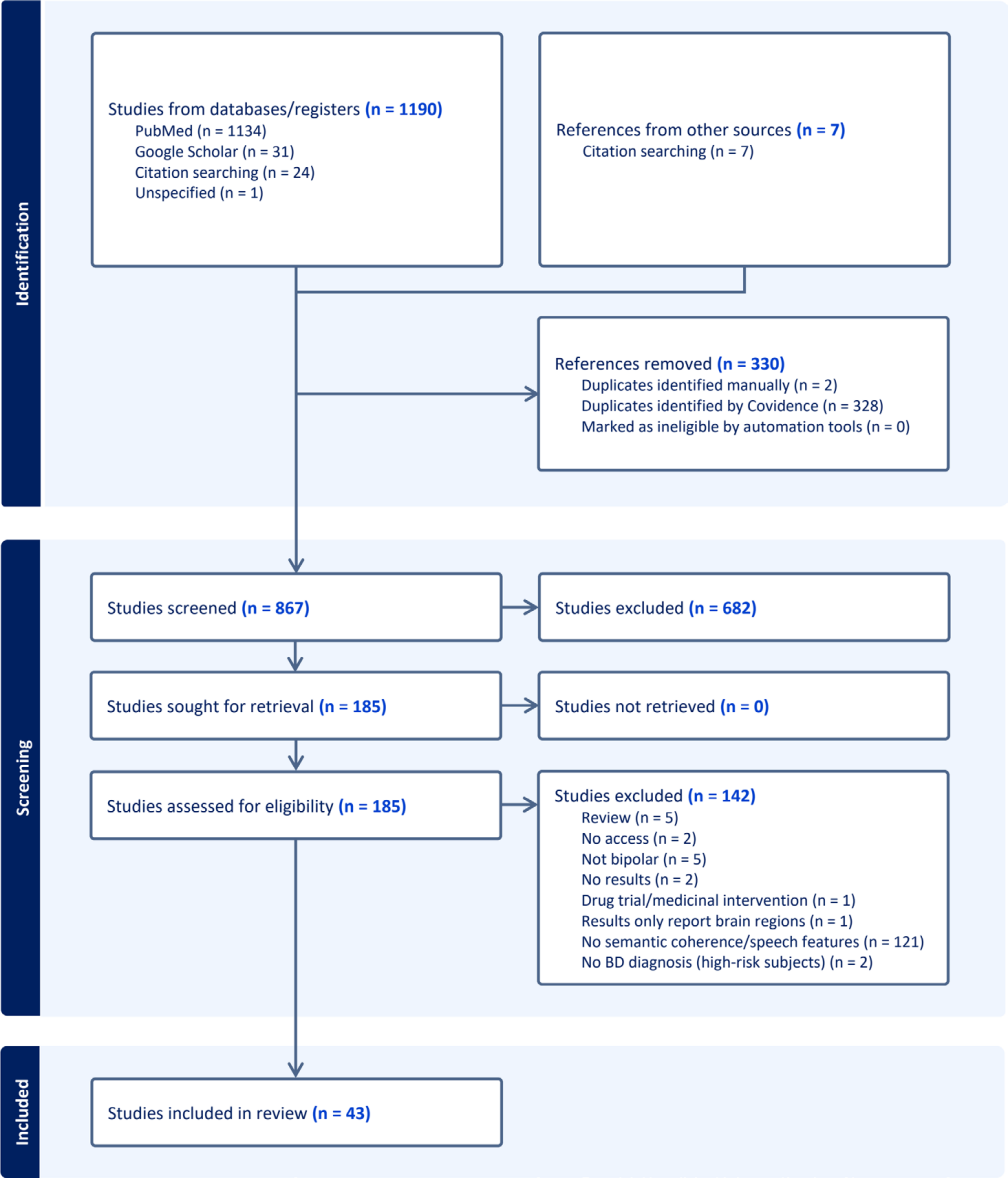
PRISMA flow diagram.

We undertook a systematic search of the literature (as per our pre‐registration [39]) using the Preferred Reporting Items for Systematic reviews and Meta‐Analyses (PRISMA) to identify all published human studies written in English pertaining to using speech markers in bipolar speech during any mood state (see Figure 1) [40]. An extension was applied upon request to include studies published until February 2026 which is a deviation from the pre‐registered protocol date of June 2024. We searched MEDLINE (via PubMed) and Google Scholar databases in February 2026. Search terms included a combination of keywords and Medical Subject Headings (MeSH) terms related to bipolar disorder and speech/language. For example, in PubMed we used the following combination: (“Natural Language Processing” [tiab] OR “Speech” OR “verbal” AND (Bipolar OR mania OR manic) NOT review). Variations of these terms were also used to ensure no relevant hits were missed. F.Z., J.A., and A.V. conducted the literature search and independently used Covidence (Veritas Health Innovation, Melbourne) to screen titles and abstracts against the inclusion criteria after removing duplicates. We used the following inclusion criteria: (1) Studies on human subjects with bipolar disorder and mean age above 18 compared to healthy or psychiatric control groups or within‐subject mood‐state comparisons; (2) Measured and analyzed spoken and/or transcribed speech; and (3) articles published in English. Full texts of relevant studies were assessed for eligibility. We then added further studies to the pool by screening the bibliography and hand‐searching all citations received by the identified studies via Google Scholar. A consensus among independent reviewers was required to determine the feasibility of including each study. Data extraction was performed by one reviewer and subsequently checked by another to ensure accuracy. Any conflicts during the selection or extraction process were resolved through a consensus meeting.

We excluded non‐empirical studies (case reports/case series). Additionally, studies focused on high‐risk subjects without a diagnosed bipolar disorder; studies reporting verbal outputs that were either restricted (e.g., scripted conversations) or likely to have been edited after production (e.g., written reports and social media texts), studies not testing language production but only comprehension or recall were not eligible. Studies with retraction notices were also excluded.

### 2.2 Topic Analysis

From the included papers, we extracted the text on introduction/background, methods, results, and discussion from abstracts. We created word clouds per section, to visually assess if the content aligned with the purpose of our review. We then applied thematic analysis to the abstracts using topic modeling with Non‐negative Matrix Factorization (NMF) to study the time‐varying semantic trends in this field. Preprocessing involved applying a Term Frequency‐Inverse Document Frequency (TF‐IDF) vectorizer limited to 1000 terms to retain only the most informative words while excluding common stopwords. The NMF model was then applied to the TF‐IDF matrix to extract latent topics, with the number of topics set to six based on an exploratory assessment of coherence and distinctiveness, balancing interpretability with adequate coverage of thematic variation in the abstracts. Each topic was represented by its top five keywords, reflecting the primary themes in the abstracts. To analyze temporal patterns in topic distribution, abstracts were grouped by publication year into 5‐year intervals (e.g., 2000–2004, 2005–2009) which ensured sufficient data density within each period.

### 2.3 Data Extraction

We extracted the following variables from the full text of each study: design (case–control, longitudinal); sample size (patients/controls); mean age of the samples, % of females in each patient cohort; mood states of subjects (euthymic/manic/depressive); mode of data acquisition (i.e., phone data, in‐person interviews, video calls); nature of speech tasks (e.g., picture description, social conversations, clinical interview, list generation); the software used for automated language analysis (e.g., openSMILE, PRAAT); major variables of interest (e.g., pause length, semantic similarity); machine learning algorithms used and their performance (if applicable). Additionally, the key variables for BD or BD state detection were extracted. Acoustic variables were grouped based on the review by Low et al. [41] into four categories: source, filter, spectral, and prosodic/melodic features. Semantic variables were grouped in seven categories: lexical, syntactic, coherence, content, pragmatic, affective, and speech graph features, a division based on key variables. The quality of included studies was assessed (see Table 1 for quality rubrics), with each study's total quality score recalculated to a score between 0% and 100%.

**TABLE 1:** Included research papers, divided in acoustic and lexical/semantic categories by speech features used.

Acoustic analysis													
Study	Design	Mood state	Sample size	Speech task	Software	Acoustic category	Results	ML classifiers	Accuracy/AUC for BD vs controls	Mean (SD) age of BD	% females in BD	Key variables	Total quality score
Vanello et al., 2012	Case‐control	Hypomania and depression	BD: 6	Reading and picture description task		Source, Prosodic	Voice pitch higher during hypomanic state compared to euthymic, and higher in euthymic over depressed. Pitch and jitter differences between mood states, except for one subject. Trends differed between subjects with some unclear mood states and characterization in a small sample.	N/A	N/A	N/A	16.67%	Pitch, jitter	38
Karam et al., 2014	Longitudinal	Hypomania	BD: 6	Naturalistic phone calls	OpenSMILE,VAD	Source, Filter, Spectral, Prosodic	Hypomania and depression can be differentiated from euthymia using speech‐based classifiers trained on structured (clinical) and unstructured cell phone recordings.	Support vector machine (SVM)	0.81 AUC (states within BD)	41(11.2)	66%	pitch, ZCR, energy, voiced intervals	63
Grünerbl et al., 2015	Longitudinal	Mania, Euthymia, depressed	BD: 10	Sensory information from smartphones (audio, accelerometer, phone calls…)	OpenSMILE	Source, Filter, Spectral, Prosodic	Automatic features effectively detected depressive, euthymic, and manic states and identified early state transitions when combining sound, location, and accelerometer data.	Naïve‐Bayes, Linear Discriminant Analysis, k‐nearest neighbor, decision trees	70% accuracy (states within BD)	N/A	91.67%	Energy, MFCC, ZCR	88
Guidi et al., 2015	Case‐control	Hypomania, mixed, euthymia and depression	BD: 9 HC: 10	Reading and picture description task		Source, Prosodic	Significant differences in audio frequency intervals were observed in hypomanic/euthymic and depressed/euthymic states, but not between healthy controls and euthymic participants.	N/A	N/A	36	100%	Voiced intervals, LTAS, pitch	50
Osmani, 2015	Longitudinal	Mania, hypomania, Euthymia, mild depression, Depression	BD: 12	Sensory information from smartphones (audio, accelerometer, phone calls…)		Prosodic	Prosodic features successfully differentiated depressive, manic, and euthymic states. Combining phone calls, location, and accelerometer data detected early mental state changes and transitions.	Naïve‐Bayes, k‐nearest neighbor, decision trees (J48), Multivariate Gaussian models	70% accuracy (states within BD)	N/A	91.67%	Pitch, speech rate, voice emotion detection	88
Abdullah et al., 2016	Longitudinal	Not specified	BD: 7	Sensory information from smartphones (audio, accelerometer, phone calls…)	Speech features	Prosodic	Conversation frequency predicted stable or unstable social rhythm	Support Vector Regression (SVR) and SVM	85% accuracy (states within BD)	N/A	71.42%	Intensity, regularity, duration of conversation	63
Faurholt‐Jepsen et al., 2016	Longitudinal	Mania, mixed, euthymia and depression	BD: 28	Naturalistic phone calls	OpenSMILE	Source, Filter, Spectral, Prosodic	Voice features more accurate, sensitive and specific in the classification of manic or mixed states compared to depression classification.	Random forest	0.89 AUC (states within BD)	30.3 (9.3)	65.40%	Pitch, MFCC, energy	100
Gideon et al., 2016	Case‐control	Mania, hypomania, mixed, euthymia and depression	BD: 37	Clinical phone calls		Spectral, Prosodic	Differentiation is possible between euthymic and symptomatic moods using clinical phone calls	SVM	0.75 AUC (states within BD)	N/A	N/A	Speech rhythm features	100
Maxhuni et al., 2016	Longitudinal	Hypomania, euthymia, mild depression, depression	BD: 5	Sensory information from smartphones (audio, accelerometer, phone calls…)		Spectral, prosodic	Automatic prosodic and spectral features successfully differentiated depressive, manic, and euthymic states. Longer pauses and slower speech rates were linked to depression, shorter and faster to mania, but mood states were not predicted	SVM, Random forest, Naïve Bayes, decision trees (C4.5)	81.8% accuracy (states within BD)	N/A	90%	Pitch, MFCC, spectral flux	88
Zhang et al., 2018	Case‐control	Mania	Mania: 30	Clinical phone calls		Source, Filter, Spectral, Prosodic	F1, F2, LPC higher in BD than HC. F4 and LPC in manic state over remission state.	N/A	N/A	41.40(11.44)	53.33%	Vowel space features, MFCC, LPCC	90
Pan et al., 2018	Longitudinal	Mania, euthymia	Mania: 21	Clinical phone calls		Filter, Spectral, Prosodic	SVM effective to discriminate manic state from euthymic state in single but not multiple patients. GMM higher detection accuracy than SVM for multiple patients in manic state detection. Speech based classifiers can differentiate between manic and euthymic mental states.	SVM, GMM	72.27 accuracy (states within BD)	34.52(15.32)	66.66%	Pitch, Vowel space features, LPC	63
Aldeneh et al., 2019	Longitudinal	Mania, euthymia, depressed	BD: 47	Naturalistic phone calls and clinical phone calls	COMBO‐SAD	Prosodic	Combining dialogue and rhythm features improves depression detection with SVM, while rhythm features alone best detect mania. Dialogue features aid depression prediction but not mania.	SVM, DNN, LR, LMEM	AUC 0.764 (states within BD)	N/A	72.34%	Speech rhythm features, dialogue features	50
Huang et al., 2020	Case‐control	Euthymia	BD: 15	Response to emotional videos	OpenSMILE	Source, Filter, Spectral, Prosodic	Using acoustics, bottlenecking, and emotion recognition, MDD was distinguished from BD.	LASM	73.33% accuracy (BD vs unipolar depression)	45.8 (10.37)	66%	MFCC, ZCR, energy	20
Yamamoto et al., 2020	Case‐control	Depression	BD: 68 MDD: 84 HC: 71	Naturalistic speech task		Prosodic	Unipolar depression had longer response times than bipolar depression. Speech rate, pause and response time correlated with depression severity. No significant differences between MDD and BD for speech rate and pauses. No significant speech features between BD and HC.	N/A	N/A	55.1(17)	55.88%	Speech rate, pause time, response time	60
Faurholt‐Jepsen et al., 2021	Longitudinal	Mania, hypomania, mixed, euthymia and depression	BD: 121	Naturalistic phone calls	OpenSMILE	Source, Filter, Spectral, Prosodic	Within BD voice features could discriminate between manic and euthymic mental states, and between bipolar depressive, unipolar depressive and euthymic mental states.	Random forest	0.79 AUC (BD vs HC)	35.71(12.35)	60%	Pitch, MFCC, energy	90
Baki et al., 2022	Case‐control	Mania, hypomania, euthymia	BD: 46 HC: 49	Picture description, Negative and positive emotion eliciting tasks	openSMILE, eGeMAPS, LIWC, TF‐IDF, BERT, GPT‐2, GPT‐3, NLTK, VADER	Source, Filter, Spectral, Prosodic	Mania is linked to high vocal and kinesthetic energy. Jitter and shimmer are tied to depression. Manic patients use incoherent discourse with heavy religious terminology.	Kernel Extreme Learning Machine	64.8 Unweighted Average Recall (states within BD)	N/A	N/A	Jitter, energy, MFCC	75
Faurholt‐Jepsen et al., 2022	Case‐control	Euthymia, depression	BD: 121	Naturalistic phone calls	OpenSMILE	Source, Filter, Spectral, Prosodic	Within BD, voice features could discriminate between manic and euthymic mental states, and between bipolar depressive, unipolar depressive and euthymic mental states.	Random forest, k‐Nearest neighbors	0.58 AUC (BD vs UD)	35.7(12.3)	60%	Pitch, MFCC, energy	80
Ji et al., 2024	Longitudinal	Mania, Depression, Remission	Manic: 20 Depressive: 39 Remission: 34	Free speech (Mood diary)	Wav2vec 2.0	Spectral, Filter, Source, prosody	Signal‐to Noise ratio can differentiate remission from manic and depressive mood states. Extracted acoustic features significantly differentiate mood states. The Chinese‐speech‐pretrain GRU achieved the best overall performance in classifying modd states (80.20%)	Linear Discriminant Analysis (LDA) GRU (Gate Recurrent Unit), BiLSTM (Bi‐directional Long Short‐Term Memory)	80.20% accuracy	Manic: 29.68 (8.03) Depressive: 29.74 (12.04) Remission: 42.05 (9.14)	Manic: 55.00% Depressive: 48.72% Remission: 79.42%	Signal to Noise Ratio, speech length/duration, Mel spectogram, MFCC	75
Kaczmarek‐Majer et al., 2024	Longitudinal	Mania, hypomania, mixed, euthymia and depression	BD: 51	Naturalistic Phone calls	OpenSMILE	Prosody, Spectral, Source, Filter	Increased depression severity is associated with quieter, lower energy, less clarity, reduced spectral dynamics and longer call duration and speaking time in males. Increased manic severity scores are associated with louder more energetic and faster speech in males, while females show quieter, lower‐pitched and less clear speech. Euthymia was predicted more reliably (83%), depression showed moderate sensitivity (62%), and mania/mixed states showed lower sensitivity.	Random Forest, decision tree, penalized regression model	Accuracy: 71.0%	36	55%	Loudness, pitch, voice signal and quality, speaking time	100
Kamińska et al., 2024	Longitudinal	Mania, mixed, euthymia and depression	BD: 75 MDD:25	Picture description, Negative and positive emotion eliciting tasks, free speech, counting, memory tasks		Source, Filter, Spectral, Prosodic	Acoustic features were usefull to distinguish ill versus not ill‐episodes of participants, and could distinguish between mania, mixed states, euthymia and depression. MFCC features were most often informative for state classification	RF, KNN, Adaboost, logistic regression, multilayer perceptron	77.3% accuracy (states within BD)	41	60%	Pitch, pause time, MFCC	25
Provost et al., 2024	Longitudinal	Mania, mixed, euthymia, depression	BD: 14 HC: 2	Naturalistic phone calls	Combined lexical and acoustic features ‐ Wav2Vec2, BERT, sentiment valence	Acoustic embedding	Automatic features contributed more to the modeling of clinical meausres compared to self‐report measures. Depression symptom severity was best modeled by combining automatic features and self‐report.	Linear Mixed Effect Models (LMEM)	N/A	53.94(13.14)	62.50%	Acoustic embeddings	75
Crocamo et al., 2025	Cross‐sectional	Mania, Depression	BD: 32	Listen and recall	Praat software	Prosodic, Source	Depressive mood states showed reduced verbal output, slowed speech, fewer words, shorter phonation, longer mean intraword time, higher silence and phonation ratio and higher instability in speech patterns (jitter). Manic states showed shorter mean intraword time, less insatbility in speech patterns.	Random forest	N/A	49.60 (14.30)	50%	Word mover's distance (WMD), number of words, silence‐phonation ratio, jitter, percentage of phonation, duration of speaking time.	88
Li et al., 2025	Cross‐sectional	Mania, depression, euthymia	Manic: 19 Depressive: 15 Euthymic: 19	Structured Clinical Interviews	HuBERT, WavLM	Spectral, prosody, filter	Speech‐based models distinguished mood states accurately, achieving the highest recognition for euthymic and depressive states, and consistently lower accuracy for manic/hypomanic states.	LSTM, ECAPA‐TDNN, TFA	85.79% Weighted Accuracy	Manic: 40.0 Depressive: 25.0 Euthymic: 34.0	Manic: 42.1% Depressive: 40.0% Euthymic: 31.6%	Mel‐spectral features, HuBERT, WavLM representations.	88
Min et al., 2025	Longitudinal	Depression (moderate‐severe and mild/minimal)	BD: 92 Mod/severe depression: 57 Minimal/mild: 35	Structured Clinical Interviews	OpenSMILE, LIWC	Source, Filter, Spectral, Prosodic, Lexical, Syntactic, Content	Both acoustic and linguistic features are associated with depression severity and can be used to detect and predict depressive states within BD. Death‐related words, temporal spectral features, changes in pitch were all assocaited with depression severity.	Scikit‐learn, Lightbm, xgboost	AUC 0.762 (speech model)	Mod/severe depression: 29.28(9.53) Minimal/mild: 31.97(12.45)	76%	Spectral, prosodic, Temporal features, MFCC, psycholinguistic categories.	75

Lexical and semantic analysis													
Study	Design	Mood state in BD	Sample size	Speech task	Software	Textual category	Results	ML classifiers	Accuracy/AUC	Mean (SD) age of BD	% females in BD	Key variables marking BD/mood state	Total quality score
Lorenz and Cobb, 1952	Case control study	Mania	Mania: 10 HC: 10	Free speech (life experiences)	Verzeano Speech Analyzer and manual coding	Lexical	Manic patients showed increased speech rate, variable silences and productivity, higher use of auxiliary verbs, pronouns, verbs per adjective, and repetitiveness, but decreased use of adjectives, prepositions, and fewer different words.	N/A	N/A	N/A	N/A	Verbs, adjectives, pronouns, propositions, lexical diversity, speech rate, pause rate	30
Andreasen and Pfohl, 1976	Case control study	Mania and depression	Mania: 16 Dep: 15	Proverb interpretation and free speech (hobbies and self)	Study‐specific dictionary‐based computer analysis	Lexical; Syntactic; Content	Mania was linked to more action verbs, power and achievement words, adjectives, and concrete nouns, while depression was associated with state‐of‐being verbs, modifying adverbs, and first‐person and personal pronouns.	N/A	N/A	33(12.6)	56.25%	verbs, adjectives, syntactic complexity, content differences, pronouns	60
Wykes and Leff, 1982	Case control study	Mania, mixed, euthymia and depression	Mania: 4 SZ: 8	Free speech (clinical)	Manual coding	Lexical; Coherence	The mania group had the most cohesive ties per sentence, but no differences were found between mania and schizophrenia in individual cohesion categories.	N/A	N/A	35.5(21.9)	75%	Cohesive ties per sentence unit	50
Harvey, 1983	Case control study	Mania	Mania: 20 (10 FTD+) SZ: 20 (10 FTD+) HC: 10	Free speech (life experiences)	Manual coding	Lexical; Coherence	Significant differences were found only in thought‐disordered patients, who showed less cohesion, fewer implicit references, and more ambiguous references.	N/A	N/A	30.15(8.65)	75%	Cohesion, references	60
Ragin and Oltmanns, 1983	Case control study	Mania	Manic: 11 Dep: 6 SZ: 10 SZa: 11 HC: 9	Free speech (clinical)	Manual coding	Content; Coherence	Schizophrenic speech was the hardest to predict using the Cloze procedure, while depressive speech was the most predictable. No comparisons were made between bipolar states.	N/A	N/A	26.2(5.8)	0%	Cloze predictability score	60
Thomas et al., 1996	Case control study	Mania	Mania: 11 SZ: 38 HC: 16	Free speech (life experiences)	Brief Syntactic Analysis (Manual)	Syntactic	Manic bipolar patients had more errors of commission than schizophrenia and healthy controls, with intermediate scores on most syntactic measures and more dysfluent speech.	N/A	N/A	N/A	N/A	Errors of commission	60
Barch and Berenbaum, 1997	Case control study	Mania	Mania: 19 SZ: 21	Free speech (life experiences)	Manual coding	Lexical; Pragmatic; Syntactic	Deppressive and negative states were linked to fewer filled pauses and less verbosity, while excitement was associated with higher verbosity.	N/A	N/A	31.3(13.9)	47%	Verbosity, syntactic complexity, filled pauses	70
Lott et al., 2002	Case control study	Likely euthymic	BD: 29 MDD: 23 SZ: 47	Free speech (emotionally neutral)	Manual coding	Pragmatic; Syntactic; Coherence	Finite verbs per sentence, verbosity and depth of embedding differentiate schizophrenia from bipolar disorder but not major depressive disorder from bipolar disorder.	Linear multivariate discriminant analysis	63.6 (BD vs SZ and HC)	27.9(9.2)	41.38%	Finite verbs per sentence, verbosity, syntactic complexity	60
Perlini et al., 2012	Case control study	Depressed, euthymic, mixed or hypomanic	BD: 30 SZ: 30 HC: 30	Picture description	Manual coding	Lexical; Syntactic; Coherence; Content	Mean length of utterance differences distinguished BD from HC, and paragrammatic errors differentiated BD‐depressed from BD‐euthymic.	N/A	N/A	44.83(9.46)	63.33%	Verbosity, Paragrammatic Errors	70
Mota et al., 2014	Case control study	Likely euthymic	BD: 20 SCZ: 20 HC: 20	Free speech (dream and waking reports)	SpeechGraphs	Speech Graph	BD patients reported dream events with more recurrent, connected, dense, and clustered graph metrics than waking events. Their speech graphs were less connected (smaller LCC and LSC) and had fewer nodes, resulting in smaller and less complex graphs compared to controls.	Naïve Bayes classifier	0.722 AUC (BD vs HC)	N/A	35%	Density, diameter, shortest path, recurrence, LCC, LSC	50
Huang et al., 2016	Case control study	Unknown	BD: 15 MDD: 15	Video description	OpenSMILE, study‐specific codes for Latent Affective Structure Model (LASM)	Affective	Latent affective structure model estimating emotion likelihood achieved better accuracy than the SVM models in mood disorder detection in both Bipolar and unipolar speech	LSTM‐based classifier	73.3% accuracy (BD vs UD)	N/A	N/A	Emotion codewords, LSTM likelihood	20
Palaniyappan et al., 2019	Case control study	Euthymic	BD: 22 SZ: 34	Picture description	SpeechGraphs	Speech Graph	BD had more connected speech ‐a higher LCC ‐ than SZ	N/A	N/A	34.6(10.4)	36.36%	LCC, LSC	70
Voleti et al., 2023	Case control study	Likely euthymic	BD: 141 SCZ/SCZa: 140 HC: 22	Role‐playing task	LIWC, BERT and Stanford Parser	Lexical; Syntactic; Pragmatic; Affective; Content	Language features related to conceptualization (volition, affect, semantic coherence, appropriateness) and formulation (lexical diversity) can identify BD or schizophrenia.	Linear regression	0.730 AUC (BD vs SZ)	48.39(12.81)	49.28%	Appropriateness of responses, verbosity, lexical diversity, semantic coherence	50
Valeriano et al., 2023	Case control study	Euthymic	BD: 19 HC: 19	Picture description	Manual coding	Lexical; Syntactic; Coherence; Content	The BD group produced more cohesion errors in both oral and written modalities and fewer thematic units in the oral modality compared to controls, with minimal changes in the descriptive discourse task.	N/A	N/A	68.0(4.3)	68.42%	Global coherence, local coherence	60
Arslan et al., 2024	Case control study	Euthymic	BD: 40 FEP: 53 HC: 50	Picture description	Natural Language Toolkit (NLTK), SBERT, spaCy	Coherence; Lexical	FEBD showed increased verbosity, conjunctions, first‐person singular pronouns, longer words and sentences compared to FEP, with semantic similarity higher than HC but lower than FEP.	Random forest classifier, Stratified k‐fold Cross Validation	71.1% (BD vs HC)	21.58(4.28)	57.50%	Semantic similarity, verbosity, pronouns	70
Esen et al., 2025	Cross‐sectional	Mania, Euthymia	BD, Mania: 30 Depression: 30 HC: 30	Free speech (any topic)	General Inquirer	Content, Lexical	The mania group showed higher values for self, natural world, ideal value, religion compared to the depression group, and had higher values for medical, death, religion and self values, and lower academic‐related speech compared to HC.	N/A	81% (across all diagnostic groups)	38.80 (12.93)	57%	Thematic category	60
Jo and Joo, 2025	Cross‐sectional	Likely euthymic	BD: 52 SCZ: 48 HC: 48	Short essay (past week), sentence completion test	KoBERT, KoGPT‐2 (korean version)	Lexical, Content, Coherence	BD group showed lower sentence perplexity (greater linguistic acceptability/predictability), greater hierarchical structure and semantic coherence compared to SCZ, and mild reductions in semantic organization compared to HC. BD differed signficantly from SCZ in semantic network structure (less disorganized) but did not differ in perplexity/predictability compared to controls.	N/A	N/A	27.5 (8.71)	59.62%	Sentence perplexity (PPL), Semantic word network metrics (degree assortavity), Global Reaching Centrality (GRC)	30
Mueller et al., 2025	Cross‐sectional	Likely euthymic	BD: 19 MDD: 49 SCZ: 19 HC: 70	Picture description	Manual Coding	Pragmatic; Coherence; Content	Negative correlation between the proportion of figurative language use and severity of FTD in BD.	N/A	N/A	44.16 (12.78)	73.68%	Metaphors, Idioms, TLI	50
Mülfarth et al., 2026	Cross‐sectional	Likely euthymic	BD: 27 MDD: 119 SCZ: 48 HC: 178	Picture description	SpaCy	Lexical; Syntactic; Coherence; Content	Highest simple sentence ratio in BD compared to SCZ and HC groups.	EBICglasso	N/A	44.55 (12.15)	62.96%	TLI, simple‐sentence ratio, syntactic complexity	50

Abbreviations: AUC, area under the curve; BD, bipolar disorder; DNN, deep neural network; DSM, diagnostic and statistical manual of mental disorders; EBICglasso, extended bayesian information criterion graphical least absolute shrinkage selection operator; ECAPA‐TDNN, emphasized channel attention, propagation, and aggregation in time delay netowrks; eGeMAPS, extended geneva minimalistic acoustic parameter set; EMA, ecological momentary assessment; FEP, first‐episode psychosis; FTD, formal thought disorder; GMM, gaussian mixture model; GPT, generative pre‐trained transformer; HAMD, hamilton depression rating scale; HC, healthy controls; ICD, international classification of diseases; KNN, k‐nearest neighbors; LASM, latent affective structure model; LCC, largest connected component; LIWC, linguistic inquiry and word count; LMEM, linear mixed effects model; LPC, linear predictive coding; LPCC, linear predictive cepstral coefficients; LR, logistic regression; LSC, largest strongly connected component; LSTM, long short‐term memory; LTAS, long‐term average spectrum; MDD, major depressive disorder; MFCC, mel‐frequency cepstral coefficients; ML, machine learning; NLTK, natural language toolkit; RDC, research diagnostic criteria; RF, random forest; SBERT, sentence‐BERT; SD, standard deviation; SVM, support vector machine; SVR, support vector regression; SZ, schizophrenia; SZa, schizoaffective disorder; TFA, time frequency attention; TF‐IDF, term frequency‐inverse document frequency; UD, unipolar depression; VAD, voice activity detection; VADER, Valence Aware Dictionary and sEntiment Reasoner; ZCR, zero‐crossing rate.

## 3 Results

### 3.1 Study Selection

**FIGURE 2:**
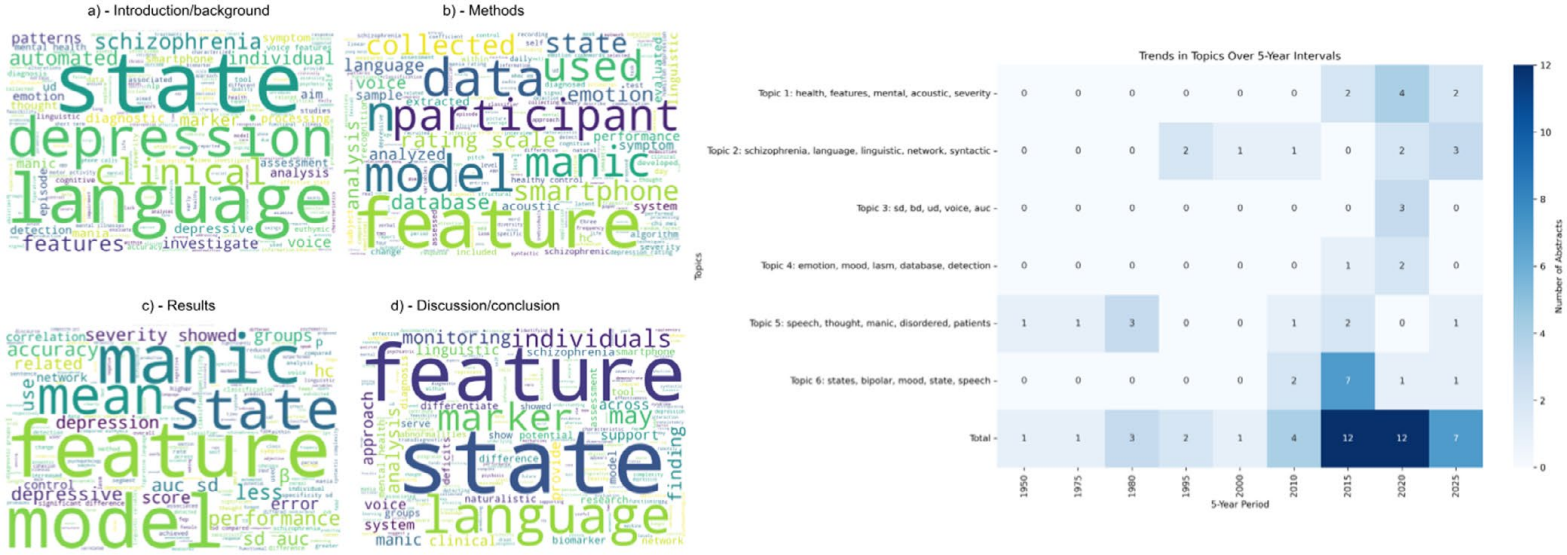
Analysis of abstract word usage and topics. Left: Wordcloud illustration of most prevalent terms present in abstracts, divided in sections: (a) introduction/background; (b) methods; (c) results, and (d) discussion/conclusion. Note that some common words including those we used as search terms were filtered out from the abstracts; these included “patient,” “patients,” “bipolar,” “disorder,” “disorders,” “BD,” “mood,” “manic,” “group,” “methods,” “results,” “speech,” “objective,” “study,” “conclusion,” “using,” and “based.” Right: Bipolar‐speech abstracts per 5‐year bin, grouped per topic derived from t‐Distributed Stochastic Neighbor Embeddings (tSNE). Total publications at the bottom row. Note that the data for the 2020–2025 column does not include papers published after June 2024. AUC, area under the curve; BD, bipolar disorder; SD, standard deviation; UD, unipolar depression.

Figure 1 shows the PRISMA flowchart of our systematic study search. From the initial screening of 867 studies, 43 were selected investigating speech in patients with BD. As shown in Figure 2, the topic analysis confirmed that the selected papers aligned with our primary focus, that is, analysis of speech features pertaining to detection or measurement of mood states in bipolar disorder or the mood states thereof.

A heatmap, also shown in Figure 2, illustrates the trend over the years, demonstrating a focus on thought, emotion, voice, syntax, speech features, and comparisons to schizophrenia. Of these, an increased focus on speech features in relation to mood is notable in recent years. Initial papers had a focus on syntactic or linguistic features such as verbs and pronouns, while papers since 2015 focused relatively more on acoustics and machine learning (with key terms such as “features,” “speech,” and “area under the curve” indicating classification algorithms).

### 3.2 Study Characteristics

Sample sizes of BD participants ranged from 4 [42] to 141 [43]. Of the 43 included studies, 14 had a longitudinal component (ranging between 2 days and 16 months of speech data collection); the rest were cross‐sectional. Some studies acquired multiple recordings but only analyzed cross‐sectional results [44, 45]. 18 studies compared BD to healthy controls, 13 with schizophrenia, and 12 with depression. With respect to the mood states captured in BD, 28 (65%) included manic or hypomanic, 21 (49%) included depressed, 27 (63%) included euthymic, and 9 (21%) included mixed states (as many included multiple states, the sum exceeds 100%). The quality of the identified studies varied notably between 20% and 100% in our scoring scheme, see Table 2.

**TABLE 2:** Quality Assessment item description and scores.

Quality indicator	Description	Scoring criteria (0–2 points)
1. Case definition	Is the BD diagnosis clearly defined using standard criteria (DSM‐5, ICD, RDC)?	2: Standard diagnostic criteria used (e.g., DSM‐5/ICD/RDC). 1: Self‐report or unclear. 0: No definition.
2. Control group	Are controls well‐defined and matched (e.g., similar demographic characteristics—age, sex, education, and language profile)?	2: Matched on age/sex, education and language profile 1: Matched on less than 2 aspects 0: Not adequate matching (1 or no matched features)
3. Outcome assessment (language)	Are validated methods used to measure language comprehension/production?	2: Validated rater‐scored tests or replicable automated approaches used. 1: Custom or non‐validated tests whose scores depend on the rater's definitions 0: No description.
4. Representativeness of patients	Are the cases representative of the wider population (e.g., all eligible cases included)?	2: All eligible cases included. 1: Partial inclusion: only hospitalized patients or only those with specific symptoms selected (e.g., FTD+ only, or males only) 0: No clear description.
5. Mood states captured	Does the study capture all 3 states of mania, depression, and euthymia in those diagnosed with BD?	2: At least 2 mood states are represented in BD samples 1: Only one state captured 0: No information on mood states provided

Abbreviations: BD, bipolar disorder; DSM‐5, Diagnostic and Statistical Manual of Mental Disorders, 5th edition; FTD, formal thought disorder; ICD, International Classification of Diseases; RDC, research diagnostic criteria.

To distinguish BD from other diagnoses and healthy controls, or different BD states from each other, 58% of studies (25/43) used classification algorithms (6 using lexico‐semantic information; 19 using acoustic features). The most common algorithms used were Support Vector Machine (6 studies) and Random Forest (8 studies). Only two studies investigated subclinical changes in mental states [46]; however, none predicted upcoming state changes.

### 3.3 Reported Observations

The extracted data with a summary of results from all 43 studies can be found in Table 3. 24 studies (*n* = 999 participants; percentage of women ranging from 16.7% [47] to 100% [48] with a mean age ranging from 30.3 to 55.1 years old) focused on acoustic (including paralinguistic) features [44, 45, 46, 47, 48, 49, 50, 51, 52, 53, 54, 55, 56, 57, 58, 59, 60, 61, 62]. 19 studies (*n* = 535 participants; percentage of women ranging from 0% to 75%, with a mean age between 21.7 and 68.0 years old) focused on lexical‐semantic features (e.g., semantic coherence, word type frequencies, and sentiment analysis) [42, 43, 63, 64, 65, 66, 67, 68, 69, 70, 71, 72, 73, 74, 75]. Manual coding procedures were more common among lexico‐semantic studies.

**TABLE 3:** Key features to detect BD or states within BD.

	Name of feature	Explanation	Number of studies	Studies using the measures
Acoustic features	Pitch	Measures variations in the fundamental frequency of speech, reflecting emotional tone, prosody, and vocal control	11	Vanello et al., 2012; Karam et al., 2014; Guidi et al., 2015; Maxhuni et al., 2016; Osmani, 2015; Faurholt‐Jepsen et al., 2016; Pan et al., 2018; Faurholt‐Jepsen et al., 2021; Faurholt‐Jepsen et al., 2022; Kaczmarek‐Majer et al., 2024; Kamińska et al., 2024
	Mel‐Frequency Cepstral Coefficients (MFCC)	MFFCs captures the spectral properties of speech, providing a representation of vocal tract characteristics	11	Grünerbl et al., 2015; Faurholt‐Jepsen et al., 2016; Maxhuni et al., 2016; Zhang et al., 2018; Huang et al., 2020; Faurholt‐Jepsen et al., 2021; Baki et al., 2022; Faurholt‐Jepsen et al., 2022; Ji et al., 2024; Kamińska et al., 2024; Min et al., 2025
	Energy	Measures the amplitude of the speech signal, reflecting the intensity or loudness of speech and is often used to analyze vocal effort and emphasis.	7	Karam et al., 2014; Grünerbl et al., 2015; Faurholt‐Jepsen et al., 2016; Huang et al., 2020; Faurholt‐Jepsen et al., 2021; Baki et al., 2022; Faurholt‐Jepsen et al., 2022
Semantic features	Verbosity	The amount of speech produced, including the length of utterances, overall word count, and tendency to provide detailed or in some cases excessive information	5	Barch and Berenbaum, 1997; Lott et al., 2002; Perlini et al., 2012; Voleti et al., 2023; Arslan et al., 2024
	Pronouns	Words used to replace nouns and refer to participants in discourse (e.g., first‐person pronouns like "I", third‐person pronouns like "they").	3	Lorenz and Cobb, 1952; Andreasen and Pfohl, 1976; Arslan et al., 2024
	Syntactic complexity	The structural intricacy of sentences, measured by features like the number of embedded clauses, sentence depth, and the variety of syntactic constructions used	4	Andreasen and Pfohl, 1976; Barch and Berenbaum, 1997; Lott et al., 2002; Mülfarth et al., 2026

*Note:* Most common features used in previous research and their explanation, divided for the acoustic and semantic domain.

### 3.4 Distinguishing BD From Other Disorders

Lexico‐semantic features differentiated speech in BD from healthy individuals and patients with schizophrenia, but comparisons between bipolar mood states per se were limited. Most studies compared euthymic BD with other groups (healthy controls, SCZ, MDD); this approach assessed between‐group differences but not within‐BD differences. Various combinations of less coherent semantic clustering, wider vector angles, increased verbosity, and semantic fluency performance distinguished bipolar disorder from the other groups. Grouping of variables following Low et al. (2020) [41] showed that for the acoustic domain, the prosodic category (pauses, speech rate, pitch) was investigated the most, in 23 out of 24 studies, followed by the spectral domain (MFCCs) for 11 out of 24 studies. For the semantic domain, lexical features (specific word usage such as pronouns or verbs) were the most common group at 12 out of 19 studies, followed by syntactic (language structure) and coherence (flow of content across words and sentences), both used in 8 papers. For investigating noncategorical but individual key features in BD, see Table 2. Our results show more overlap in measures used in the acoustic compared to the semantic domain, both for categories and key variables.

### 3.5 Detecting and Predicting States Within BD

Acoustic features were most effective in differentiating between: (1) manic and euthymic states (for instance Faurholt‐Jepsen et al. [52]; Pan et al. [59]); (2) manic/hypomanic and depressed states (for instance Guidi et al. [48]; Maxhuni et al. [57]); and (3) manic, mixed, and depressed states [44, 53]; for full results see Table 3. While speech‐based features, such as jitter and pitch, were able to distinguish between the presence or absence of manic/depressed states (or in the case of Osmani [58], detecting state change), none of the studies reported on features that tracked changes in symptom severity or predicted a forthcoming relapse or switch.

### 3.6 Classification

Results of classifying BD from other groups ranged from an area under the curve (AUC) of 0.58 (distinguishing BD from MDD [46]) to an accuracy of 73% (distinguishing BD from healthy controls [64]). Classification or detection of BD states ranged from an AUC of 0.63 [45] to an AUC of 0.89 [52].

## 4 Discussion

To our knowledge, this is the first systematic review to examine markers of speech in individuals with BD. The studies we identified confirm the emerging interest in the use of speech features to study mood states in BD and report several observable linguistic patterns, specifically in the acoustic and lexical‐semantic domains across bipolar mood states.

We note that (1) distinct speech patterns can be recovered from individuals with BD compared to healthy controls and people with schizophrenia or MDD in some studies. Across many examined variables, the degree of impairment in the speech of individuals with BD occupies an intermediate position: being less severe than schizophrenia but also not as typical as healthy controls. This finding suggests a potential “ladder of severity” in speech abnormalities, positioning BD between healthy controls and schizophrenia [43, 66, 72, 73, 74, 76]. We note a tendency to use schizophrenia as the most common psychiatric comparator, with no studies contrasting BD and other diagnoses that are potential comorbidities (e.g., ADHD, PTSD, borderline personality, or substance‐induced mood states), highlighting a key research gap.

We also note that (2) the capacity of speech and language features to identify different states of BD. Acoustic parameters appear to have reasonable success in discriminating between manic/mixed states and depressive states in BD. Among non‐acoustic features, derailment, increased grammatical errors, word repetition, and verb use were associated with the manic state, while increased use of personal pronouns, modifying adverbs, and poorer verbal fluency related to the depressive state. These differential patterns also contributed to statistical separation between unipolar and bipolar depressed youth (who had more pressured speech) and between hypomanic and depressed mood states within BD.

However, for (3) predicting impending mood states, the current state of evidence linking speech patterns with BD mood states is marred by a notable lack of longitudinal, within‐subject examinations. Only 14 (33%) of studies in our review attempted repeated measurements in relation to mood states (see for instance Faurholt‐Jepsen et al. [52]; Pan et al. [59]), More recently, Kaczmarek‐Majer et al. [77] demonstrated that acoustic features can predict BD phases, achieving approximately 71% accuracy in classifying euthymia, depression and manic/mixed states/However, prediction of *upcoming* mood state changes, rather than concurrent classification, remains undemonstrated. Next to individual prediction, longitudinal, within‐individual design also reduces inter‐individual confounding effects [51] and allows evaluation of the individual predictive value of speech markers. These and other notable research gaps were appraised using Miles' Gap Framework (2017) [78] (see Table 4).

**TABLE 4:** Gap framework for the study of speech markers in bipolar disorder.

Gap domain	Contributing observations	Key questions/opportunities
Knowledge gap (Absence of evidence)	Lack of longitudinal studies on euthymic individuals that predict upcoming mood states.	Can speech‐NLP features in euthymic states predict and differentiate upcoming state shifts/relapses? Can the use of speech markers avert relapses in BD/prevent misdiagnosis of bipolar disorder and MDD?
Evidence gap (Contradictory observations)	There are notable variations in the reported ability to (1) classify BD from UD using acoustic markers (AUC 0.58 to 73% accuracy) and (2) differentiate SCZ and BD with lexico‐semantic features.	Can the use of more specific NLP tools improve the ability to differentiate between bipolar mental states? What are the most sensitive/specific speech features that can reduce the discrepancy in the findings?
Empirical gap (Insufficient evaluations)	Many classification/prediction analyses lack external validation. Variable selection lacks theoretical motivation, leading to a lack of multilevel evaluations (e.g., combined lexical, semantic, and acoustic variables).	Can collaborative shared speech databases be created to make external validation more feasible? What theoretical considerations/psychometric evaluations can guide variable selection for mood state predictions and trait‐based diagnostic classifications?
Population gap (representative samples)	Some speech markers are more sensitive than others to the social background (race/ethnicity, cultural background, socioeconomic status) as well as medication use. Subgroups of unmedicated patients (e.g., untreated youth), multilingual patients, other diagnostic groups (e.g., borderline personality), and immigrants are not studied to date.	Does medication affect speech? Is there a difference between drug‐naive/first‐episode manic/hypomanic patients and those who have been on medication for several years? Do age, immigrant status, native language, and other comorbid diagnoses affect speech markers in BD?
Practical knowledge gap (Action: knowledge divide)	None of the studies we appraised included qualitative components to ascertain the acceptability and preferences of people with lived experience of BD.	Are the research protocols and devices used for speech acquisition suitable for clinical use? What patient‐level barriers prevent the integration of speech‐based diagnostic tools into routine care? How much can we generalise the current observations to real‐world clinical contexts?
Methodological gap (Method/research design gaps)	A wide variety of speech elicitation protocols are used. There is no consensus on study methods and sets of variables examined and reported details. Bias scores vary notably, with no standardisation of reporting across speech‐NLP studies.	Can the research community reach a consensus on the optimal speech elicitation and processing procedures for various clinical use cases? What features are required to meet the minimum reporting standards of speech‐NLP studies in BD?

Abbreviations: AUC, area under the curve; BD, bipolar disorder; NLP, natural language processing; UD, unipolar depression.

A promising development in recent times is the emergence of studies, for example, the PRIORI project [79] and CALIBER [80], as well as the Kaczmarek‐Majer et al. [77] study, that are leading these efforts. Notably, Kaczmarek‐Majer et al. [77] built on the methodological framework of Faurholt‐Jepsen et al. [52], adopting their cutoff thresholds and time‐windowing approaches (Kaczmarek‐Majer et al. [77], Faurholt‐Jepsen et al. [52]). However, such methodological continuity remaines the exception rather than the norm. Several acquisitions and analysis approaches are being used without external validation for data‐driven classification studies, with variables of interest not being selected on the basis of common theoretical or psychometric considerations. Sharing datasets and methodologies across research groups will promote collaboration and accelerate progress in the field. The representativeness of the BD samples studied to date is also poor; sociodemographic characteristics (age, parental socioeconomic status, education, native language status, and sex [8]), substance use [81], and treatment exposure [82] may influence speech features, but these are not explicitly accounted for in most studies. Similarly, integrating multiple analytic levels—semantic, acoustic, and temporal—will provide a richer understanding of speech changes linked to mood states.

Until these issues are addressed, speech markers are not ready as a clinical tool for relapse prediction or diagnostic decisions in BD. However, the state‐related acoustic and lexico‐semantic patterns identified in this literature suggest a near‐term role as an aid to clinical assessment, particularly when repeated speech samples can be compared within individuals over time (Hansen et al. [83]; Gumus et al. [84]). Such approach may help clinicians detect subtle emerging hypomanic or mixed features that are frequently missed, and prompt earlier, more targeted assessment (Anmella et al. [80]). Clinically, this could be the most valuable in the intervals between scheduled psychiatric appointments during which mood state changes can go undetected until they become severe (Cerimele and Fortney, [85]). Passive, smartphone‐based speech collection, as employed in Kaczmarek‐Majer et al. [77] and the PRIORI project, offer a feasible and relatively unobtrusive means of continuing monitoring in naturalistic settings (Kaczmarek‐Majer et al. [77], Ann Arbor M., [79]). Any clinical deployment will require prospective validation to false alarms, and safeguards for privacy and consent.

Our work has several strengths, including the pre‐registration of our search, methodical bias assessment of individual studies, and topic analysis to synthesize emerging trends. Various limitations must also be considered when interpreting our observations. We limited our search to published English language reports and adult samples (age > 18). We were not able to undertake a quantitative synthesis (meta‐analysis) due to the notable methodological heterogeneity among studies. Nonetheless, based on the lack of consensus regarding variable selection and acquisition protocols, we do not expect more homogenous observations to emerge from unpublished studies or studies published in other languages. A program of research that addresses the issues raised in Table 4 may lend itself to a quantitative synthesis in the future.

We note a potential for speech markers to aid in three key areas of need in bipolar disorder: (1) distinguishing BD from other disorders; (2) identifying different BD states, such as euthymia and mania; and (3) predicting impending mood states. While there is ample evidence supporting the pursuit of this line of work, the predictive value for each of the above clinical applications remains untested. Future research, particularly longitudinal studies, and concerted community efforts are needed to position speech assessments as a key approach in the study of BD.

## Funding

This work was supported by Brain and Behavior Research Foundation, Canada First Research Excellence Fund, Canadian Institutes of Health Research and Fonds de Recherche du Québec—Santé.

## Data Availability

The data that support the findings of this study are available from the corresponding author upon reasonable request.
